# Homme à utérus ou syndrome de la persistance des canaux de Müller (PMDS)

**Published:** 2012-11-29

**Authors:** Khalid Khattala, Youssef Bouabdallah

**Affiliations:** 1 Service de chirurgie pédiatrique, CHU Hassan II, Fès, Maroc

**Keywords:** Syndrome, persistance des canaux de Müller, pseudo-hermaphrodisme, génotype, testostérone, syndrome, persistence of Müllerian ducts, pseudohermaphroditism, genotype, testosterone

## Images en medicine

Le syndrome de la persistance des canaux de Müller (PMDS) est une forme rare de pseudo-hermaphrodisme masculin, caractérisé par la présence d'un utérus et les trompes de Fallope en raison de l’échec derégression du conduitmullerien chez les mâles du génotype normal. Le syndrome est causé soit par une quantité insuffisante du facteur anti mullerien facteur ou en raison de l'insensibilité de l'organe cible à ce facteur. Le diagnostic de PMDS est souvent mis en place au cours du traitement opératoire des anomalies associées comme la hernie inguinale et cryptorchidie. C'un syndrome qui se transmis sur le mode autosomique récessif. Nous rapportons le cas d'un enfant de sexe masculin âgé de 6 ans, admis pour ectopie testiculaire bilatérale non palpable à l'examen. Le caryotype est 46 XY, le taux de testostérone est normal. L'exploration coelioscopique a objectivé la présence d'un utérus avec des trompes et des testicules, on a proposé un abaissement en deux temps selon la technique de Fowler Stephens.

**Figure 1:**
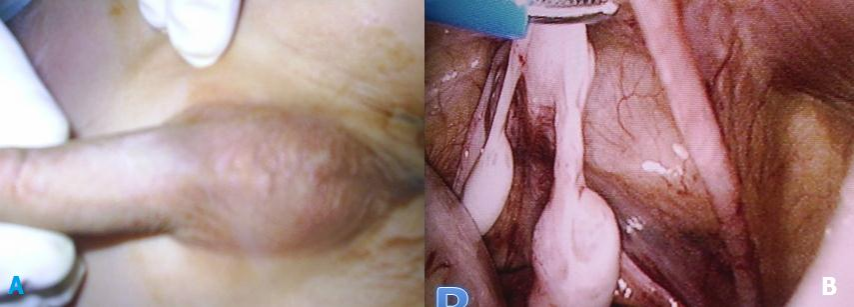
A: Garçon avec ectopie testiculaire bilatérale non palpable; B: aspect coelioscopique montrant un utérus avec des trompes et des testicules

